# Association of provider advice and gestational weight gain in twin pregnancies: a cross-sectional electronic survey

**DOI:** 10.1186/s12884-020-03107-3

**Published:** 2020-07-23

**Authors:** Kara M. WHITAKER, Meghan BARUTH, Rebecca A. SCHLAFF, Christopher P. CONNOLLY, Jihong LIU, Sara WILCOX

**Affiliations:** 1 Department of Health and Human Physiology and Department of Epidemiology, University of Iowa, E116 Field House, Iowa City, IA 52242 USA; 2 Department of Health Sciences, Saginaw Valley State University, University Center, MI USA; 3 Department of Educational Leadership, Sports Studies, and Educational/Counseling Psychology, Washington State University, Pullman, Washington, USA; 4 Department of Epidemiology and Biostatistics, University of South Carolina, Columbia, SC USA; 5 Department of Exercise Science, University of South Carolina, Columbia, SC USA

**Keywords:** Patient-provider communication, Health care provider, Gestational weight gain, Pregnancy, Twins

## Abstract

**Background:**

Little is known about patient-provider communication on gestational weight gain among women pregnant with twins, a growing population at high risk for adverse maternal and neonatal outcomes. We examined if women’s report of provider advice on gestational weight gain was consistent with the Institute of Medicine (IOM) weight gain guidelines for twin pregnancies, and the association of provider advice on weight gain with women’s weight gain during their twin pregnancy.

**Methods:**

We conducted a cross-sectional survey of 276 women who delivered twins and received prenatal care in the United States. The 2009 IOM provisional weight gain guidelines for twin pregnancies defined whether provider advice on weight gain and women’s weight gain were below, within, or above guidelines. Multinomial logistic regression examined associations between provider advice on weight gain with women’s weight gain, after adjustment for maternal age, gestational age at delivery, education, parity, twin type, use of assisted reproductive technologies and pre-pregnancy BMI category.

**Results:**

Approximately 30% of women described provider advice on weight gain below the IOM guidelines, 60% within, and 10% above guidelines. Compared to women who reported weight gain advice within guidelines, women who reported advice below guidelines or who reported no advice were 7.1 (95% CI: 3.2, 16.0) and 2.7 (95% CI: 1.3, 5.6) times more likely to gain less than recommended, respectively. Women who reported provider advice above guidelines were 4.6 (95% CI: 1.5, 14.2) times more likely to exceed guidelines.

**Conclusions:**

Provider advice on gestational weight gain may be an important predictor of women’s weight gain during twin pregnancies, highlighting the critical need for accurate provider counseling to optimize health outcomes.

## Background

Over the last three decades, the twin birth rate in the United States has risen nearly 80%, accounting for 1 in every 30 births in 2015 [1, 2]. Compared to singleton pregnancies, twin gestations are associated with a higher risk of adverse neonatal outcomes, including fetal death, preterm birth, low birth weight, and intrauterine growth restriction [2, 3]. Women pregnant with twins are also at greater risk for health complications, with higher rates of gestational diabetes, hypertensive disorders, anemia, postpartum hemorrhage, and cesarean deliveries as compared to women with singleton pregnancies [4–6]. While there are non-modifiable factors that contribute to the increased risk of infant and maternal morbidity in twin pregnancies (i.e. maternal age, parity, prior medical history), appropriate gestational weight gain (GWG) is increasingly recognized as an important modifiable factor contributing to positive maternal and infant health outcomes [7].

In 2009, the Institute of Medicine (IOM) released updated GWG guidelines, including provisional guidelines for women pregnant with twins. Normal weight women pregnant with twins are recommended to gain 37–54 lbs. (17–25 kg), overweight women 31–50 lbs. (14–23 kg) and obese women 25–42 lbs. (11–19 kg) [7]. These guidelines reflect the 25th and 75th percentile range of total weight gain among women who delivered twins weighing ≥2500 g on average at 37–42 weeks gestation. Women with GWG below the IOM guidelines for twin pregnancies are at increased risk for preterm delivery [8–10] and small for gestational age infants [10–14], while GWG within or above guidelines is associated with normal birth weight [9, 12, 15, 16]. Limited evidence also suggests that women with weight gain above guidelines during twin pregnancies are at higher risk for maternal complications, including gestational diabetes, pregnancy-induced hypertension, preeclampsia, or anemia (aOR 1.63, 95% CI: 1.02–2.60) and cesarean delivery (aOR 1.85, 95% CI: 1.20–2.87) [17].

To achieve optimum pregnancy outcomes, the American College of Obstetricians and Gynecologists (ACOG) recommends that health care providers counsel their pregnant patients on appropriate weight gain [18]. Growing evidence suggests that among women with singleton pregnancies, provider advice during prenatal care may be an important determinant of weight gain during pregnancy [19–23]. However, little is known about health care provider advice related to GWG for women with twin pregnancies. Given that women with multiple fetuses share a disproportionate burden of poor maternal and fetal outcomes compared to singleton pregnancies, it is important to develop a better understanding of patient-provider communication on GWG during twin pregnancies, including whether provider advice is associated with greater adherence to the IOM weight gain guidelines. Therefore, the aims of this study are to: 1) determine the prevalence of provider advised weight gain consistent with IOM guidelines for twin pregnancies, and 2) examine the association of provider advice on GWG with women’s GWG during twin pregnancies.

## Methods

### Study population

Women in the Mothers of Twins Health Study were recruited in May, 2018 using social media sites targeting mothers of multiples. A brief description of the study and link to the screening form was posted on several websites (e.g., La Leche League for Mothers of Multiples). Women were eligible for the study if they met the following self-reported criteria: aged 18–44 years, twin birth within the last three years, first prenatal visit prior to 16 weeks gestation, received prenatal care in the United States, knowledge of twin gestation before the third trimester, and not currently pregnant. Women who met eligibility criteria were invited to complete a cross-sectional internet-based survey assessing health behaviors as well as health care provider advice on weight gain, physical activity, and diet during their twin pregnancy (see Supplementary File 1 for study survey) [24]. This paper reports findings related to GWG. A $10 Amazon gift card was provided to women who completed the full survey. Written informed consent was obtained from all participants and study protocols were approved by the University of Iowa Institutional Review Board.

### Exposure: provider advice on gestational weight gain

Participants were asked if a health care provider (e.g. doctor, midwife, nurse) discussed how much weight they should gain during their twin pregnancy (yes, no, not sure). Individuals who selected yes were then asked how much total weight in pounds their health care provider recommended they gain using an open-ended response. Mean values were calculated for women who specified a range of weight gain (e.g. 30–40 lbs. coded as 35 lbs). Participants were also asked which healthcare provider(s) discussed GWG during their twin pregnancy (Ob/Gyn, Maternal Fetal Medicine Specialist, Infertility Specialist, Nurse Practitioner, Nurse, Dietician, Other).

### Outcome: Women’s gestational weight gain

Participants reported their total weight gain (lbs) during their twin pregnancy. Assessment of total GWG occurred prior to assessment of provider advice on GWG to limit social desirability bias.

### Personal history questionnaire

Height and pre-pregnancy weight were ascertained by self-report and used to calculate pre-pregnancy body mass index (BMI; kg/m^2^). Pre-pregnancy BMI was categorized as underweight/normal weight (< 25.0 kg/m^2^), overweight (25.0–29.9 kg/m^2^), or obese (≥30.0 kg/m^2^). Underweight women (*n* = 6) were included with normal weight women because the IOM does not specify GWG guidelines for underweight women pregnant with twins. While underreporting of weight has been previously reported, particularly among non-pregnant overweight or obese women [25], self-reported pre-pregnancy weight during pregnancy has been demonstrated as reliable and valid [26, 27]. Additional measures included: maternal age at twin delivery, race, marital status, education, employment status, household income, parity prior to the twin pregnancy, use of assisted reproductive technologies for their twin pregnancy (yes/no), twin pregnancy type (dichorionic/diamniotic, dichorionic/monoamniotic, monochorionic/monoamniotic), pregnancy complications (gestational diabetes, high blood pressure, hypertension, preeclampsia, anemia, twin to twin transfusion syndrome, and hyperemesis gravidarum), smoking status and alcohol consumption during the twin pregnancy, and gestational age at delivery.

### Statistical analysis

Descriptive analyses, including frequencies and means, for key variables were conducted. Differences in sociodemographics and pregnancy characteristics, stratified by provider advice on GWG (yes/no), and separately, by women’s GWG category, were assessed using independent samples t-tests, chi-square tests, or fisher’s exact test, as appropriate. The IOM guidelines for twin pregnancies defined whether women’s report of provider advised weight gain and women’s self-reported weight gain were compliant with these guidelines [7]. Based on pre-pregnancy BMI categories, provider GWG advice was categorized into the following groups: below, within, or above IOM guidelines. Because women’s GWG differs based on gestational age at delivery, a rate of GWG per week was calculated. The IOM guidelines for twin pregnancies were developed for women undergoing delivery at or following 37 weeks gestation, therefore the lower and upper bound of the IOM guidelines were divided by 37 to estimate GWG per week. The rate of weight gain for underweight/normal weight, overweight, and obese patients was calculated as 1.00–1.46 lbs./week (0.45–0.66 kg/week), 0.84–1.35 lbs./week (0.38–0.61 kg/week), and 0.68–1.14 lbs./week (0.31–0.52 kg/week), respectively. Similarly, women’s GWG per week was calculated by dividing total GWG by gestational age at delivery, and classified as below, within, or above IOM guidelines using this value. This method is the most commonly used approach for assessing compliance to IOM guidelines for twin pregnancies [8, 9, 12, 28]. However, this method assumes a constant rate of weight gain across all pregnancy trimesters, and does not account for a slower expected rate of weight gain in the first trimester [29]. To address this limitation, we used a secondary approach [14], assuming an average cumulative GWG in the first trimester of a twin pregnancy of 7.9 lbs. (3.6 kg) for underweight/normal weight women, 4.6 lbs. (2.1 kg) for overweight women, and 4.4 lbs. (2.0 kg) for obese women, as specified by the IOM [7]. Second and third trimester recommended weekly GWG rates for each BMI group were calculated using the following formula: (IOM recommended total GWG – IOM average cumulative GWG in first trimester) / (37 weeks – 13 weeks), or 1.21–1.92, 1.10–1.89, and 0.86–1.57 lbs./week (0.55–0.87, 0.50–0.86, 0.39–0.71 kg/week) for underweight/normal, overweight, and obese women, respectively. Women were classified as gaining weight below, within, or above IOM guidelines based on each woman’s second and third trimester weekly GWG rate, using a similar approach.

Associations between provider recommended weight gain (below IOM guidelines, within IOM guidelines, above IOM guidelines, or not advised on GWG) and women’s adequacy of GWG (below IOM guidelines, within IOM guidelines, or above IOM guidelines using the two described methods) were assessed using multinomial logistic regression models. Models were adjusted for maternal age, gestational age at delivery, education, parity, twin type (dichorionic/diamniotic vs. dichorionic/monoamniotic or monochorionic/monoamniotic), assisted reproductive technologies (yes/no), and pre-pregnancy BMI category. All statistical analyses were conducted using SAS 9.4, (SAS Institute, Inc., Cary, NC).

## Results

**Fig. 1:**
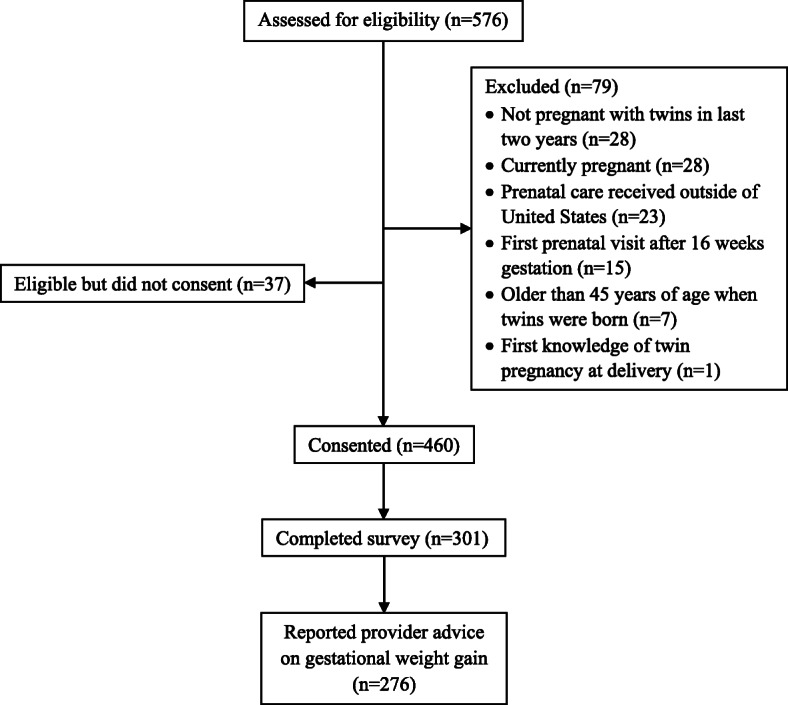
Mothers of Twins Health Study Participant Flow Chart

As seen in Figs. 1, 576 women were assessed for eligibility. Seventy-nine women were excluded for not meeting the eligibility criteria, and 37 women who met inclusion criteria did not consent to take part in the study. A total of 460 participants consented and began the survey, with 301 completing the full survey (52% of those screened for eligibility and 65% of those who consented). Fifteen women did not report provider advice on GWG (yes/no) and 10 women reported receiving provider advice on GWG but did not quantify the recommended amount and were excluded from analyses for a final analytic sample of 276. Women who were excluded were more likely to be nulliparous prior to the twin pregnancy than those who were included (68.0% vs. 44.6%, *p* = 0.025); no other differences in participant characteristics were observed.

Participant characteristics, overall and stratified by provider advice on GWG (yes/no), are presented in Table 1. On average, participants were aged 31.5 ± 4.0 years (range 21–43 years) at delivery and completed the survey 11.3 ± 7.7 months postpartum (63.4% < 12 months postpartum, 29.4% 12–24 months postpartum, 7.3% > 24 to < 36 months postpartum). The majority of women were white and married with high levels of education. Nearly 40% used assisted reproductive technologies for their twin pregnancy, and the majority of twins were dichoroinoic/diamniotic (lowest risk twin category). A high percentage of participants reported one or more pregnancy complications (59.1%), including gestational diabetes, high blood pressure or hypertension, preeclampsia, anemia, twin to twin transfusion syndrome, and/or hyperemesis gravidarum. Average length of gestation was 35.8 ± 2.1 weeks. Using the weekly rate of GWG across all trimesters, 26.8% of women gained weight below the IOM guidelines, 46.7% within guidelines, and 26.5% above guidelines. This was nearly identical when using the alternate approach, assuming a fixed GWG in the first trimester and calculating a weekly rate of GWG across the second and third trimesters, with 25.7% gaining below IOM guidelines, 46.4% within guidelines, and 27.9% above guidelines (data not shown). Only seven women changed IOM categories using the alternate approach, with three transitioning from below to within guidelines and four transitioning from within to exceeding guidelines.

**Table 1:** Participant characteristics, overall and stratified by provider advice on gestational weight gain (*N* = 276)

Participant Demographics	Total N = 276	Advised on GWG N = 188	Not Advised on GWG ***N*** = 88	p-value^a^
Age at delivery, mean years ± SD	31.5 ± 4.0	31.2 ± 4.1	32.0 ± 3.8	0.141
Time since delivery, mean months ± SD	11.3 ± 7.8	11.4 ± 7.7	11.0 ± 7.8	0.646
Race (*N* = 275), n(%)				0.560
White	262 (95.3)	177 (94.7)	85 (96.6)	
Other^b^	13 (4.7)	10 (5.4)	3 (3.4)	
Marital status, n(%)				0.322
Married	257 (93.1)	177 (94.2)	80 (90.9)	
Unmarried	19 (6.9)	11 (5.9)	8 (9.1)	
Education, n(%)				0.387
Some college or less	53 (19.2)	40 (21.3)	13 (14.8)	
Bachelor’s degree	120 (43.5)	79 (42.0)	41 (46.6)	
Master’s degree	81 (29.4)	52 (27.7)	29 (33.0)	
Professional or Doctorate degree	22 (8.0)	17 (9.0)	5 (5.7)	
Employment, n(%)				**0.007**
Employed full time	126 (45.7)	97 (51.6)	29 (33.0)	
Employed part time	40 (14.5)	21 (11.2)	19 (21.6)	
Homemaker	110 (39.9)	70 (37.2)	40 (45.5)	
Household income (*N* = 263), n(%)				0.817
< $50,000	35 (13.3)	24 (13.6)	11 (12.8)	
$50,000–$99,999	89 (33.8)	61 (34.5)	28 (32.6)	
$100,000–$149,999	81 (30.8)	56 (31.6)	25 (29.1)	
> $150,000	58 (22.1)	36 (20.3)	22 (25.6)	
Parity prior to twin pregnancy, n(%)				0.061
Nulliparous	123 (44.6)	91 (48.4)	32 (36.4)	
Multiparous	153 (55.4)	97 (51.6)	56 (63.6)	
Pre-pregnancy BMI Category, n(%)				0.937
Underweight/normal weight	139 (50.4)	96 (51.1)	43 (48.9)	
Overweight	66 (23.9)	44 (23.4)	22 (25.0)	
Obese	71 (25.7)	48 (25.5)	23 (26.1)	
Use of assisted reproductive technologies, n(%)	103 (37.3)	72 (38.3)	31 (35.2)	0.623
Smoking in twin pregnancy, n(%)	4 (1.5)	3 (1.6)	1 (1.1)	0.999
Alcohol use in twin pregnancy, n(%)	9 (3.3)	7 (3.7)	2 (2.3)	0.723
Twin pregnancy type, n(%)				0.944
Dichorionic/diamniotic	222 (80.4)	151 (80.3)	69 (80.2)	
Monochorionic/diamniotic or monochorionic/monoamniotic	54 (19.6)	37 (19.7)	17 (19.8)	
Pregnancy complications^c^, n(%)	163 (59.1)	106 (56.4)	57 (64.8)	0.187
Gestational age at delivery, mean weeks ± SD	35.8 ± 2.1	35.7 ± 2.1	35.9 ± 2.1	0.494
GWG, mean lbs. ± SD	40.6 ± 15.9	40.3 ± 15.2	41.3 ± 17.5	0.616
Adequacy of GWG^d^, n(%)				0.561
Below IOM guidelines	74 (26.8)	48 (25.5)	26 (29.6)	
Within IOM guidelines	129 (46.7)	92 (48.9)	37 (42.1)	
Above IOM guidelines	73 (26.5)	48 (25.5)	25 (28.4)	

Abbreviations: GWG = gestational weight gain, SD = standard deviation, BMI = body mass index, IOM = Institute of Medicine

^a^P-value calculated using independent samples t-tests, chi-square tests, or fisher’s exact test, as appropriate. Bolded values are statistically significant (*p* < 0.05)

^b^Black or African American, American Indian or Alaska Native, Asian, Pacific Islander, and Other

^c^Gestational diabetes, high blood pressure or hypertension, preeclampsia, anemia, twin to twin transfusion syndrome, and hyperemesis gravidarum

^d^Using weekly rate of GWG across all trimesters

Approximately 68% of women reported receiving provider advice on GWG during their twin pregnancy (*N* = 188). Women who reported provider advice on GWG were more likely to be employed full time compared to those who did not report provider advice on GWG (*p* = 0.007). There were no other significant differences across participant characteristics by provider advice on GWG. Women primarily reported receiving GWG advice from their Ob/Gyn (60.5%) or Maternal Fetal Medicine Specialist (19.9%). There were also no significant differences in participant characteristics when stratifying by women’s GWG category (below, within, or above IOM guidelines using weekly rate of GWG across all trimesters, see Table 2), with the exception of total GWG increasing across IOM categories (*p* < 0.001).

**Table 2:** Participant characteristics, stratified by women’s self-reported gestational weight gain^a^ (N = 276)

Participant Demographics	GWG Below IOM Guidelines (***N*** = 74)	GWG Within IOM Guidelines (***N*** = 129)	GWG Above IOM Guidelines (***N*** = 73)	p-value^b^
Age at delivery, mean years ± SD	31.6 ± 3.6	31.1 ± 4.2	32.1 ± 4.2	0.234
Time since delivery, mean months ± SD	10.1 ± 6.8	11.2 ± 7.7	12.6 ± 8.6	0.146
Race (*N* = 285), n(%)				0.761
White	70 (94.6)	121 (94.5)	71 (97.3)	
Other^c^	4 (5.4)	7 (5.5)	2 (2.7)	
Marital status, n(%)				0.234
Married	71 (96.0)	121 (93.8)	65 (89.0)	
Unmarried	3 (4.1)	8 (6.2)	8 (11.0)	
Education, n(%)				0.870
Some college or less	13 (17.6)	25 (19.4)	15 (20.6)	
Bachelor’s degree	30 (40.5)	61 (47.3)	29 (39.7)	
Master’s degree	24 (32.4)	33 (25.6)	24 (32.9)	
Professional or Doctorate degree	7 (9.5)	10 (7.8)	5 (6.9)	
Employment, n(%)				0.574
Employed full time	32 (43.2)	64 (49.6)	30 (41.1)	
Employed part time	11 (14.9)	20 (15.5)	9 (12.3)	
Homemaker	31 (41.9)	45 (34.9)	34 (46.6)	
Household income, n(%)				0.846
< $50,000	11 (15.3)	18 (14.8)	6 (8.7)	
$50,000–$99,999	21 (29.2)	41 (33.6)	27 (39.1)	
$100,000–$149,999	23 (31.9)	37 (30.3)	21 (30.4)	
> $150,000	17 (23.6)	26 (21.3)	15 (21.7)	
Parity prior to twin pregnancy, n(%)				0.058
Nulliparous	28 (37.8)	54 (41.9)	41 (56.2)	
Multiparous	46 (62.2)	75 (58.2)	32 (43.8)	
Pre-pregnancy BMI Category, n(%)				0.770
Normal weight	37 (50.0)	69 (53.5)	33 (45.2)	
Overweight	17 (23.0)	31 (24.0)	18 (24.7)	
Obese	20 (27.0)	29 (22.5)	22 (30.1)	
Use of assisted reproductive technologies, n(%)	25 (33.8)	52 (40.3)	26 (35.6)	0.613
Smoking in twin pregnancy, n(%)	2 (2.7)	0 (0.0)	2 (2.7)	0.079
Alcohol use in twin pregnancy, n(%)	0 (0.0)	6 (4.7)	3 (4.1)	0.169
Twin pregnancy type, n(%)				0.556
Dichorionic/diamniotic	62 (83.8)	104 (80.6)	56 (76.7)	
Monochorionic/diamniotic or monochorionic/monoamniotic	12 (16.2)	25 (194)	17 (23.3)	
Pregnancy complications^d^, n(%)	45 (60.8)	72 (55.8)	46 (63.0)	0.569
Gestational age at delivery, mean weeks ± SD	35.8 ± 2.4	35.8 ± 2.0	35.7 ± 2.1	0.952
GWG, mean lbs. ± SD	23.3 ± 9.3	40.1 ± 7.4	59.0 ± 11.5	**< 0.001**
Provider advice on GWG, n(%)				0.561
Advised on GWG	48 (64.9)	92 (71.3)	48 (65.8)	
Not advised on GWG	26 (35.1)	37 (28.7)	25 (34.3)	

Abbreviations: GWG = gestational weight gain, IOM = Institute of Medicine, SD = standard deviation, BMI = body mass index

^a^Using weekly rate of GWG across all trimesters

^b^P-value calculated using independent samples t-tests, chi-square tests, or fisher’s exact test, as appropriate. Bolded values are statistically significant (*p* < 0.05)

^c^Black or African American, American Indian or Alaska Native, Asian, Pacific Islander, and Other

^d^Gestational diabetes, high blood pressure or hypertension, preeclampsia, anemia, twin to twin transfusion syndrome, and hyperemesis gravidarum

As seen in Table 3, of those who reported receiving provider advice on GWG and quantified the amount of weight gain recommended (*N* = 188), approximately 30% of women reported provider advice below IOM guidelines, 60% within guidelines, and 10% above guidelines. There were no differences in provider recommended GWG by pre-pregnancy BMI category (*p* = 0.623).

**Table 3:** Women’s report of provider advised gestational weight gain, overall and stratified by pre-pregnancy body mass index (N = 188)^a^

		Pre-pregnancy BMI Category		
Provider Advised GWG	Total (N = 188)	Normal weight (***N*** = 96)	Overweight (***N*** = 44)	Obese (***N*** = 48)
Below IOM guidelines	56 (29.8)	27 (28.1)	16 (36.4)	13 (27.1)
Within IOM guidelines	113 (60.1)	58 (60.4)	26 (59.1)	29 (60.4)
Above IOM guidelines	19 (10.1)	11 (11.5)	2 (4.6)	6 (12.5)

Abbreviations: BMI = body mass index, GWG = gestational weight gain, IOM = Institute of Medicine

^a^Women who reported no provider advice on GWG (n = 88) are excluded from table. Data presented as N(%)

Associations of provider advice on GWG with women’s compliance to the IOM guidelines for twin pregnancies, using weekly rate of GWG across all trimesters, are shown in Table 4**.** Compared to women who reported GWG advice within IOM guidelines, women who reported advice below guidelines or who reported no advice were 7.1 (95% CI: 3.2, 16.0) and 2.7 (95% CI: 1.3, 5.6) times more likely to gain less than recommended by the IOM, respectively. Women who reported provider advice above the IOM guidelines were 4.6 (95% CI: 1.5, 14.2) times more likely to exceed the IOM guidelines. Study findings were similar when assuming a fixed GWG in the first trimester and calculating weekly rate of GWG across the second and third trimesters (see **Supplementary File 2**).

**Table 4:** Association of provider advice with women’s compliance to the Institute of Medicine guidelines, using weekly rate of GWG across all trimesters (*N* = 276)

	GWG Below IOM Guidelines		GWG Above IOM Guidelines	
Provider Advised GWG	Adjusted OR^a^	95% CI	Adjusted OR^a^	95% CI
Below IOM guidelines	**7.11**	**3.15, 16.03**	1.56	0.62, 3.92
Above IOM guidelines	0.64	0.07, 5.81	**4.58**	**1.48, 14.19**
Within IOM guidelines	Reference	Reference	Reference	Reference
Did not discuss	**2.67**	**1.28, 5.59**	1.82	0.90, 3.69

Abbreviations: GWG = gestational weight gain, IOM = Institute of Medicine

^a^Model adjusted for maternal age at delivery, education, parity, twin type (dichorionic/diamniotic vs. dichorionic/monoamniotic or monochorionic/monoamniotic), assisted reproductive technologies (yes/no), and pre-pregnancy BMI category. Bolded values are statistically significant (*p* < 0.05)

## Discussion

In this cross-sectional internet-based survey of women who delivered twins within the last two years, nearly 70% of participants reported receiving provider advice on GWG during their twin pregnancy. To our knowledge, no prior studies have examined provider advice on GWG in twin pregnancies; however, previous studies of women pregnant with singletons found that only 29–52% of women reported provider counseling on GWG [20, 21, 23, 30], which is lower than observed in the present study. While concerning that nearly one-third of women did not recall provider advice on GWG during their twin pregnancy, it appears that GWG discussions may occur more frequently in women pregnant with twins compared to women pregnant with singletons.

Of those participants who reported provider advice on GWG and quantified the amount of weight gain recommended, approximately 30% reported provider advice below the provisional IOM guidelines for twin pregnancies, 60% within, and 10% above guidelines (i.e. 40% gave recommendations outside of IOM guidelines). In studies of singleton pregnancies, accuracy of provider recommended weight gain compared to IOM guidelines varies widely, with anywhere from 29 to 85% of women reporting provider GWG advice within IOM guidelines [20, 23, 30, 31]. A particularly concerning finding in the present study is the relatively high percentage of women reporting provider advice on weight gain below the IOM guidelines for twin pregnancies (30%) as compared to singleton pregnancies (2–16%) [20, 23, 31–33]. The risks associated with inadequate GWG in singleton pregnancies are well established; women who gain below the IOM guidelines are at increased risk for infant mortality, preterm birth, small-for-gestational age and intrauterine growth restriction [7, 34, 35]. Though less is known about the risks associated with inadequate GWG in twin pregnancies, data suggest women pregnant with twins may be at an increased risk for aforementioned complications [2, 3]. As a result, it is especially critical that health care providers are knowledgeable about the IOM guidelines for twin pregnancies and counsel their patients accordingly, with emphasis placed on the importance of adequate weight gain.

Women who reported provider advice below IOM guidelines were 7.1 times more likely to experience inadequate weight gain, while women who reported advice above IOM guidelines were 4.6 times more likely to experience excessive weight gain. Earlier studies of singleton pregnancies also provide supportive evidence indicating that provider advice on GWG is associated with women’s GWG [19, 20, 22, 32]. For example, using data from the Los Angeles Mommy and Baby study, Liu and colleagues found that compared to women reporting provider GWG advice within IOM guidelines, those who reported advice below guidelines were 1.7 times (95% CI: 1.3, 2.2) more likely to have inadequate weight gain [19]. Women in their study who reported provider advice above IOM guidelines were also 2.0 times (95% CI: 1.4, 2.9) more likely to exceed guidelines. Larger effect sizes were observed in the current study compared to others examining provider advice and GWG in singleton pregnancies. Due to their higher risk for adverse health outcomes, women pregnant with twins have more prenatal visits compared to women with singleton pregnancies. Therefore, women’s GWG is monitored more frequently and there are additional opportunities for provider counseling, which may explain in part why effect sizes are larger compared to others. It is also possible that the larger effect sizes observed in this study are the result of self-selection bias, with those agreeing to participate being more interested in weight gain and related behaviors. However, this type of bias is a concern for all studies requiring informed consent, with those who agree to take part in research differing from those who decline participation. Overall these results are promising as it appears women are listening to and following health care provider advice on GWG. It is therefore imperative that health care providers are communicating accurate information.

This study fills an important gap in the literature by examining provider advice on GWG and women’s compliance to IOM guidelines in twin pregnancies. An important strength of this study was the examination of women’s compliance to the IOM guidelines using two different approaches, taking into consideration the lower expected rate of weight gain in the first trimester compared to the second and third trimesters.

However, there are several study limitations to acknowledge. First, pre-pregnancy BMI was self-reported, and underreporting of weight may have occurred which could result in misclassification of pre-pregnancy BMI. Additionally, total GWG was assessed up to three years postpartum, and accuracy of recall may decrease over time. However, the majority of participants were < 12 months postpartum (63%), and self-reported GWG up to one year postpartum was previously found to be a reliable substitute when birth certificate GWG data are unavailable [36]. Furthermore, a study by McClure et al., reported moderate agreement between documented and self-reported gestational weight gain as a continuous variable at 4–12 years postpartum [37]. However, it is important to note that there were observed differences when categorizing GWG according to the IOM guidelines using documented versus self-reported GWG in this study. For example, 20% of women with documented excessive GWG were misclassified according to the IOM guidelines when using self-reported GWG. However, the recall period in the McClure et al., study was longer than the present study and only included women with singleton pregnancies. Given the greater risk associated with twin pregnancies, it is possible women pregnant with twins more accurately recall their GWG compared to women with singleton pregnancies, although to date no studies have examined differences in recall bias of GWG between twin and singleton pregnancies. Women with singleton pregnancies are more likely to recall GWG that is higher than their documented GWG and are thus more likely to be misclassified as having excessive GWG [38]. If this same pattern holds true in the current study, this could differentially bias associations with provider advice. Study participants also reported provider advice on GWG, and corroborating information was not available from health care providers. Future studies should verify both pre-pregnancy BMI and GWG with medical chart records, and include provider recall of conversations on GWG. An additional limitation is that this study was limited to a highly educated, predominately white population who were largely recruited from a breastfeeding support website, which limits generalizability. Previous studies examining provider advice and GWG in singleton pregnancies have been conducted in predominately white populations [20, 21, 23, 32, 33, 39–42], as done for the present study, with several exceptions where the majority of participants were Hispanic or African American women [19, 22, 43–45]. It is also known that women with twins are less likely to initiate breastfeeding compared to women with singletons [46]; thus it is important to acknowledge that the population in the present study is unique as they were largely recruited from a breastfeeding support group. However, La Leche League for Mothers of Multiples has nearly 9000 Facebook members and thus the findings are generalizable for women in this group and similar support groups. Given the limited research in twin mothers, this study should inform the development of future research using more rigorous methodological approaches in diverse populations. Furthermore, an important next step is to assess differences in awareness of IOM guidelines by provider type (e.g., nurse, midwife, Ob/Gyn) as well as examine whether associations of provider advice and GWG differ based on who is providing advice.

## Conclusion

Findings indicate that women’s report of provider advice on GWG may be an important predictor of women’s GWG during twin pregnancies. Prenatal care providers need to be made aware of the IOM provisional weight gain guidelines for women pregnant with twins and receive training on how to effectively counsel women on appropriate GWG during their pregnancy to optimize health outcomes for mother and children. However, the GWG guidelines for twin pregnancies are provisional due to the limited research in this area. As the rate of twin pregnancies continues to grow, more research is needed to better understand the health effects on the mother and children of weight gain below or above IOM guidelines, including information on the most appropriate rate of weight gain in each trimester to optimize health outcomes.

## Supplementary information

**Additional file 1: Supplementary File 1.** Mothers of Twins Health Study Survey. Cross-sectional internet-based survey assessing health behaviors and health care provider advice on weight gain, physical activity, and diet during their twin pregnancy.

**Additional file 2: Supplementary File 2.** Association of provider advice with women’s compliance to the Institute of Medicine provisional GWG guidelines for twin pregnancies, using a fixed GWG in the first trimester and weekly rate of GWG in the second and third trimesters (*N* = 276). This table displays results from multinomial regression analyses examining associations of women’s report of provider advice on GWG with women’s compliance to the provisional GWG guidelines for twin pregnancies using a secondary approach to categorize GWG. In these analyses, we used a fixed GWG in the first trimester and weekly rate of GWG in the second and third trimesters to categorize GWG as below, within, or above guidelines.

## Data Availability

The datasets used and analyzed during the current study are available in the University of Iowa’s Institutional Repository.

## Abbreviations

ACOG: American College of Obstetricians and Gynecologists
aOR: Adjusted odds ratio
BMI: Body mass index
CI: Confidence interval
GWG: Gestational weight gain
IOM: Institute of Medicine
OR: Odds ratio
