# Supplementary material for: Association of provider advice and gestational weight gain in twin pregnancies: a cross-sectional electronic survey
Source: BMC Pregnancy Childbirth. 2020 Jul 23;20:417. doi: 10.1186/s12884-020-03107-3 (PMC7376962; doi:10.1186/s12884-020-03107-3)
Supplement: Supplementary file 1 — Additional file 1: Supplementary File 1. Mothers of Twins Health Study Survey. Cross-sectional internet-based survey assessing health behaviors and health care provider advice on weight gain, physical activity, and diet during their twin pregnancy. [file 12884_2020_3107_MOESM1_ESM.pdf]

Section A: General questions about your most recent twin pregnancy

When were your twins born?

____

How many weeks pregnant were you when you delivered your twins?

____
(weeks)

How much did your FIRST-BORN twin weigh at birth?

◯ Less than 3 lbs 5 oz (1500 g)
◯ 3 lbs 5 oz - 5 lbs 7 oz (1500 - 2499 g)
◯ 5 lbs 8 oz - 8 lbs 13 oz (2500 - 4000 g)
◯ Greater than 8 lbs 13 oz (4000 g)
◯ Not sure

How much did your SECOND-BORN twin weight at birth?

◯ Less than 3 lbs 5 oz (1500 g)
◯ 3 lbs 5 oz - 5 lbs 7 oz (1500 - 2499 g)
◯ 5 lbs 8 oz - 8 lbs 13 oz (2500 - 4000 g)
◯ Greater than 8 lbs 13 oz (4000 g)
◯ Not sure

How old were YOU when your twins were born?

____
(years)

In what state were your twins born?

◯ Alabama
◯ Alaska
◯ Arizona
◯ Arkansas
◯ California
◯ Colorado
◯ Connecticut
◯ Delaware
◯ Florida
◯ Georgia
◯ Hawaii
◯ Idaho
◯ Illinois
◯ Indiana
◯ Iowa
◯ Kansas
◯ Kentucky
◯ Louisiana
◯ Maine
◯ Maryland
◯ Massachusetts
◯ Michigan
◯ Minnesota
◯ Mississippi
◯ Missouri
◯ Montana
◯ Nebraska
◯ Nevada
◯ New Hampshire
◯ New Jersey
◯ New Mexico
◯ New York
◯ North Carolina
◯ North Dakota
◯ Ohio
◯ Oklahoma
◯ Oregon
◯ Pennsylvania
◯ Rhode Island
◯ South Carolina
◯ South Dakota
◯ Tennessee
◯ Texas
◯ Utah
◯ Vermont
◯ Virginia
◯ Washington
◯ West Virginia
◯ Wisconsin
◯ Wyoming
◯ Other

Are your twins identical or fraternal?

◯ Identical
◯ Fraternal
◯ Not sure

What type of twin pregnancy did you have?

◯ Dichorinic/diamniotic (different placentas, different amniotic sacs)
◯ Monochorionic/diamniotic (same placenta, different amniotic sacs)
◯ Monochorionic/monoamniotic (same placenta, same amniotic sac)
◯ Not sure

Fertility Treatments

Were your twins conceived with the help of fertility treatments?

◯ Yes
◯ No

If YES, what type of fertility treatment did you use to get pregnant with your twins? Select all that apply.

☐ Fertility drugs (e.g. Clomid, Femara)
☐ Intrauterine insemination (IUI)
☐ In vitro fertilization (IVF)
☐ Other

If other, please explain.

Labor and Delivery

Were you induced (given medication or other intervention to begin labor)?

◯ Yes
◯ No

What type of birth did you have with your twins?

◯ Vaginal
◯ Cesarean section
◯ Vaginal and cesarean section

If vaginal birth, did you have an epidural (spinal injection to reduce pain)?

◯ Yes
◯ No

Infant Feeding

Did you breastfeed or pump breast milk to feed your twins, even for a short period of time?

◯ Yes
◯ No

If yes, how long did you breastfeed or feed pumped milk to your twins?

◯ < 1 week
◯ 1 week - 1 month
◯ 1-3 months
◯ 3-6 months
◯ 6-9 months
◯ 9-12 months
◯ >12 months
◯ I am still breastfeeding or feeding pumped milk to my twins

For the first six months after your twins were born, what primary feeding methods did you use? Select all that apply.

☐ Breast milk directly from the breast
☐ Expressed breast milk from bottle
☐ Donor breast milk
☐ Formula

Health Conditions during Pregnancy

Did a health care provider (e.g. doctor, midwife, nurse) tell you that you had any of the following conditions during your most recent twin pregnancy:

	Yes	No	Not sure
Gestational diabetes (high blood sugar that developed during pregnancy)?	◯	◯	◯
High blood pressure or hypertension?	◯	◯	◯
Preeclampsia, eclampsia, pregnancy induced hypertension, or toxemia (high blood pressure and/or protein in the urine)?	◯	◯	◯
Anemia (low red blood cell count)?	◯	◯	◯
Twin-to-twin transfusion syndrome (can only occur with identical twins that share a placenta)?	◯	◯	◯
Hyperemesis gravidarum (persistent severe vomiting)?	◯	◯	◯
Other complications?	◯	◯	◯

If yes, please explain these other complications.

____

You have completed section 1 of 7.

Please double check that you answered all questions before moving to the next section.

Section B: Weight before, during, and after your most recent twin pregnancy

If you are not sure of the answers to the questions below, please take your best guess. Enter numbers only in the space provided.

How tall are you?
For example, if you are 5 feet 4 inches, write 5 in the box labeled feet and 4 in the box labeled inches.

33) Feet

____

34) Inches

____

35) How much did you weigh in pounds just BEFORE you became pregnant with twins (before gaining any pregnancy weight)?

____
(pounds)

36) How much total weight in pounds did you gain DURING your twin pregnancy?

____
(pounds)

37) How much did you weigh in pounds SIX MONTHS AFTER you delivered your twins?
Please skip this question if less than six months postpartum.

____
(pounds)

38) How much do you CURRENTLY weigh in pounds?

____
(pounds)

You have completed section 2 of 7.

Please double check that you answered all questions before moving to the next section.

Section C: Physical activity before and during your most recent twin pregnancy

Please read the following statement before answering the next set of questions.

We are interested in how much moderate or vigorous physical activity you did before and during your twin pregnancy.

MODERATE ACTIVITY causes small increases in breathing or heart rate.
Examples: brisk walking, bicycling, vacuuming, gardening, or anything else that causes small increases in breathing or heart rate

VIGOROUS ACTIVITY causes large increases in breathing or heart rate.
Examples: running, aerobics, heavy yard work, or anything else that causes large increases in breathing or heart rate

BEFORE Pregnancy

BEFORE you became pregnant with your twins, in a typical week, did you do any moderate or vigorous activities for at least 10 minutes at a time?

◯ Yes
◯ No

BEFORE you became pregnant with your twins, how many DAYS PER WEEK did you do these activities for at least 10 minutes at a time?

	0	1	2	3	4	5	6	7
Moderate activity days/week	◯	◯	◯	◯	◯	◯	◯	◯
Vigorous activity days/week	◯	◯	◯	◯	◯	◯	◯	◯

BEFORE you became pregnant with your twins, on days when you did these activities for at least 10 minutes at a time, how many total MINUTES PER DAY did you spend doing these activities?

	None	10-20	20-30	30-40	40-50	50-60	>60
Moderate activity minutes/day	◯	◯	◯	◯	◯	◯	◯
Vigorous activity minutes/day	◯	◯	◯	◯	◯	◯	◯

During the FIRST TRIMESTER

During the FIRST TRIMESTER (weeks 1-12) of your twin pregnancy, did you do any moderate or vigorous activities for at least 10 minutes at a time?

◯ Yes
◯ No

During the FIRST TRIMESTER of your twin pregnancy, how many DAYS PER WEEK did you do these activities for at least 10 minutes?

	0	1	2	3	4	5	6	7
Moderate activity days/week	◯	◯	◯	◯	◯	◯	◯	◯
Vigorous activity days/week	◯	◯	◯	◯	◯	◯	◯	◯

During the FIRST TRIMESTER of your twin pregnancy, on days when you did these activities for at least 10 minutes at a time, how many total MINUTES PER DAY did you spend doing these activities?

	None	10-20	20-30	30-40	40-50	50-60	>60
Moderate activity minutes/day	◯	◯	◯	◯	◯	◯	◯
Vigorous activity minutes/day	◯	◯	◯	◯	◯	◯	◯

During the SECOND TRIMESTER

During the SECOND TRIMESTER (weeks 13-27) of your twin pregnancy, did you do any moderate or vigorous activities for at least 10 minutes at a time?

◯ Yes
◯ No

During the SECOND TRIMESTER of your twin pregnancy, how many DAYS PER WEEK did you do these activities for at least 10 minutes?

	0	1	2	3	4	5	6	7
Moderate activity days/week	◯	◯	◯	◯	◯	◯	◯	◯
Vigorous activity days/week	◯	◯	◯	◯	◯	◯	◯	◯

During the SECOND TRIMESTER of your twin pregnancy, on days when you did these activities for at least 10 minutes at a time, how many MINUTES PER DAY did you spend doing these activities?

	None	10-20	20-30	30-40	40-50	50-60	>60
Moderate activity minutes/day	◯	◯	◯	◯	◯	◯	◯
Vigorous activity minutes/day	◯	◯	◯	◯	◯	◯	◯

During the THIRD TRIMESTER

During the THIRD TRIMESTER (weeks 28-40) of your twin pregnancy, did you do any moderate or vigorous activities for at least 10 minutes at a time?

◯ Yes
◯ No
◯ I delivered my twins before the third trimester (before 28 weeks)

During the THIRD TRIMESTER of your twin pregnancy, how many DAYS PER WEEK did you do these activities for at least 10 minutes?

	0	1	2	3	4	5	6	7
Moderate activity days/week	○	○	○	○	○	○	○	○
Vigorous activity days/week	○	○	○	○	○	○	○	○

During the THIRD TRIMESTER of your twin pregnancy, on days when you did these activities for at least 10 minutes at a time, how many MINUTES PER DAY did you spend doing these activities?

	None	10-20	20-30	30-40	40-50	50-60	>60
Moderate activity minutes/day	○	○	○	○	○	○	○
Vigorous activity minutes/day	○	○	○	○	○	○	○

You have completed section 3 of 7.

Please double check that you answered all questions before moving to the next section.

Section D: Diet before and during your most recent twin pregnancy

Please read the following statement before answering the next set of questions.

A HEALTHY DIET includes plenty of fruits and vegetables, low fat dairy products, protein, fiber, and whole grains (like whole wheat breads and brown rice) instead of refined grains (like white breads and rice). A healthy diet includes watching portion sizes and avoiding eating too much of very sugary and fatty foods and drinks.

BEFORE Pregnancy

59) BEFORE you became pregnant with your twins, in general, how healthy was your overall diet?

◯ Poor ◯ Fair ◯ Good ◯ Very good ◯ Excellent

During the FIRST TRIMESTER (weeks 1-12 weeks)

60) During the FIRST TRIMESTER (weeks 1-12) of your twin pregnancy, in general, how healthy was your overall diet?

◯ Poor ◯ Fair ◯ Good ◯ Very good ◯ Excellent

61) During the FIRST TRIMESTER (weeks 1-12) of your twin pregnancy, how did the amount of food you ate compare with the amount eaten before pregnancy?

◯ A lot less food ◯ A little less food ◯ About the same ◯ A little more food ◯ A lot more food

During the SECOND TRIMESTER (weeks 13-27 weeks)

62) During the SECOND TRIMESTER (weeks 13-27) of your twin pregnancy, in general, how healthy was your overall diet?

◯ Poor ◯ Fair ◯ Good ◯ Very good ◯ Excellent

63) During the SECOND TRIMESTER (weeks 13-27) of your twin pregnancy, how did the amount of food you ate compare with the amount eaten before pregnancy?

◯ A lot less food ◯ A little less food ◯ About the same ◯ A little more food ◯ A lot more food

During the THIRD TRIMESTER (weeks 28-40 weeks)

64) During the THIRD TRIMESTER (weeks 28-40) of your twin pregnancy, in general, how healthy was your overall diet?

◯ Poor ◯ Fair ◯ Good ◯ Very good ◯ Excellent

65) During the THIRD TRIMESTER (weeks 28-40) of your twin pregnancy, how did the amount of food you ate compare with the amount eaten before pregnancy?

◯ A lot less food ◯ A little less food ◯ About the same ◯ A little more food ◯ A lot more food

You have completed section 4 of 7.

Please double check that you answered all questions before moving to the next section.

Section E: Discussions with your health care providers during your most recent twin pregnancy

Pregnancy Weight Gain

Did a health care provider (e.g. doctor, midwife, nurse) discuss with you how much weight you should gain during your twin pregnancy?

◯ Yes
◯ No
◯ Not sure

How many pounds did your health care provider recommend that you gain during your twin pregnancy?

____
(pounds)

Who started the conversation about weight gain?

◯ Me
◯ Health care provider
◯ Not sure

Which health care provider discussed how much weight you should gain during your twin pregnancy?
Select all that apply.

☐ Ob/Gyn
☐ Family Practitioner
☐ Maternal-Fetal Medicine Specialist
☐ Infertility Specialist
☐ Midwife
☐ Nurse Practitioner
☐ Nurse
☐ Dietician
☐ Other

How satisfied were you with the information that you received from your health care provider about weight gain during your twin pregnancy?

◯ Very Dissatisfied
◯ Dissatisfied
◯ Neutral
◯ Satisfied
◯ Very Satisfied

Pregnancy Physical Activity

Did a health care provider (e.g. doctor, midwife, nurse) discuss physical activity with you during your twin pregnancy?

◯ Yes
◯ No
◯ Not sure

Did a health care provider (e.g. doctor, midwife, nurse) discuss the amount of physical activity you should be getting during your twin pregnancy (e.g. minutes/day, minutes/week, days/week)?

◯ Yes
◯ No
◯ Not sure

If yes, what did your health care provider recommend for physical activity amount?

Did a health care provider (e.g. doctor, midwife, nurse) discuss types of physical activities you could participate in during your twin pregnancy (e.g. walking, jogging, swimming, yoga)?

◯ Yes
◯ No
◯ Not sure

If yes, what did your health care provider recommend for physical activity type?

Did a health care provider (e.g. doctor, midwife, nurse) discuss the intensity level or how hard you should be working while being physically active during your twin pregnancy (e.g. light-intensity, moderate-intensity, vigorous-intensity)?

◯ Yes
◯ No
◯ Not sure

If yes, what did your health care provider recommend for physical activity intensity?

Did a health care provider (e.g. doctor, midwife, nurse) discuss making changes to your physical activity as your twin pregnancy progressed?

◯ Yes
◯ No
◯ Not sure

If yes, how did the recommendations from your health care provider on physical activity change as your pregnancy progressed?

Did a health care provider (e.g. doctor, midwife, nurse) give any other advice related to physical activity that was not already asked about?

◯ Yes
◯ No
◯ Not sure

If yes, what else did your health care provider recommend about physical activity?

Who started the conversation about physical activity?

◯ Me
◯ Health care provider
◯ Not sure

Which health care provider discussed physical activity with you during your twin pregnancy?
Select all that apply.

☐ Ob/Gyn
☐ Family Practitioner
☐ Maternal-Fetal Medicine Specialist
☐ Infertility Specialist
☐ Midwife
☐ Nurse Practitioner
☐ Nurse
☐ Dietician
☐ Other

How satisfied were you with the information that you received from your health care provider about physical activity during your twin pregnancy?

◯ Very Dissatisfied
◯ Dissatisfied
◯ Neutral
◯ Satisfied
◯ Very Satisfied

Pregnancy Nutrition

Did a health care provider (e.g. doctor, midwife, nurse) discuss nutrition or healthy eating with you during your twin pregnancy?

◯ Yes
◯ No
◯ Not sure

What did your health care provider recommend?

Did a health care provider (e.g. doctor, midwife, nurse) discuss making any changes to your diet as your twin pregnancy progressed?

◯ Yes
◯ No
◯ Not sure

If yes, how did the recommendations from your health care provider on diet change as your pregnancy progressed?

Did a health care provider (e.g. doctor, midwife, nurse) discuss how many calories you should be eating during your twin pregnancy?

◯ Yes
◯ No
◯ Not sure

If yes, how many calories did your health care provider recommend you eat during your twin pregnancy?

____

Who started the conversation about nutrition/healthy eating?

◯ Me
◯ Health care provider
◯ Not sure

Which health care provider discussed nutrition/healthy eating with you during your twin pregnancy? Select all that apply.

☐ Ob/Gyn
☐ Family Practitioner
☐ Maternal-Fetal Medicine Specialist
☐ Infertility Specialist
☐ Midwife
☐ Nurse Practitioner
☐ Nurse
☐ Dietician
☐ Other

How satisfied were you with the information that you received from your health care provider about nutrition/healthy eating during your twin pregnancy?

◯ Very Dissatisfied
◯ Dissatisfied
◯ Neutral
◯ Satisfied
◯ Very Satisfied

General Health Care Provider Questions

How much do you value the opinion of the primary health care provider that you saw during your twin pregnancy?

◯ Not at all
◯ Slightly
◯ Moderately
◯ Highly

Did you see the same health care provider for the majority (over half) of your prenatal visits?

◯ Yes
◯ No

Did you attend a group practice where the health care providers rotated for your prenatal visits?

◯ Yes
◯ No

Did you change where you received prenatal care during your twin pregnancy?

◯ Yes
◯ No

You have completed section 5 of 7.

Please double check that you answered all questions before moving to the next section.

Section F: Questions related to your health

Would you say that in general your health is:

◯ Poor
◯ Fair
◯ Good
◯ Very good
◯ Excellent

In the three months BEFORE your twin pregnancy, did you smoke cigarettes every day, some days, or not at all?

◯ Every day
◯ Some days
◯ Not at all

DURING your twin pregnancy, did you smoke cigarettes every day, some days, or not at all?

◯ Every day
◯ Some days
◯ Not at all

In the three months BEFORE your twin pregnancy, how many alcoholic drinks did you have in an average week?

◯ I didn't drink
◯ Less than 1 drink a week
◯ 1 to 3 drinks a week
◯ 4 to 6 drinks a week
◯ 7 to 13 drinks a week
◯ 14 drinks a week or more

DURING your twin pregnancy, how many alcoholic drinks did you have in an average week?

◯ I didn't drink
◯ Less than 1 drink a week
◯ 1 to 3 drinks a week
◯ 4 to 6 drinks a week
◯ 7 to 13 drinks a week
◯ 14 drinks a week or more

Has a doctor, nurse, or other health professional EVER told you that you had any of the following?
Select all that apply.

☐ Hypertension or high blood pressure
☐ Heart attack (also called myocardial infarction)
☐ Stroke
☐ Diabetes - insulin dependent
☐ Diabetes - type 2 or non-insulin dependent
☐ Cancer
☐ Other serious conditions
☐ None of the above

If you selected other serious conditions, please explain.

You have completed section 6 of 7.

Please double check that you answered all questions before moving to the next section.

Section G: About you

As a reminder, all information will be kept private and will not affect any services you are now getting.

105) How old are you?

____
(years)

106) What is your marital status?

◯ Married
◯ Widowed
◯ Divorced
◯ Separated
◯ Never married
◯ Unmarried couple

107) What is the highest degree you have completed?

◯ Less than a high school diploma
◯ High school diploma or equivalency (GED)
◯ Some college, no degree
◯ Associate degree (e.g. AA, AS)
◯ Bachelor's degree (e.g. BA, BS)
◯ Master's degree (e.g. MA, MS, MEd)
◯ Professional (MD, JD, DDS, etc.)
◯ Doctorate (PhD, EdD)
◯ Other

108) How many children under the age of 18 live in your household?

◯ 0
◯ 1
◯ 2
◯ 3
◯ 4
◯ 5
◯ 6 or more

109) How many children did you give birth to BEFORE your most recent twin pregnancy?

◯ 0
◯ 1
◯ 2
◯ 3 or more

110) BEFORE your most recent twin pregnancy, did you experience any of the following pregnancy outcomes? Check all that apply.

☐ Miscarriage
☐ Stillbirth
☐ Preterm birth (birth before 37 weeks gestation)
☐ Low birthweight (baby weighed less than 5 pounds, 8 ounces)
☐ Pregnancy with multiples (twins, triplets, quads, etc.). Do not count your most recent twin pregnancy.
☐ None of the above

111) How many children did you give birth to AFTER your most recent twin pregnancy?

◯ 0
◯ 1
◯ 2
◯ 3 or more

112) Which of the following apply to you regarding your current working status? Choose all that apply.

☐ Employed for wages full time
☐ Employed for wages part time
☐ Self-employed
☐ Out of work
☐ Homemaker
☐ Student
☐ Disabled and unable to work

113) During the past 12 months, what was your total household income before taxes? Include your income, your husband or partner's income, and any other income that you may have used.

◯ Less than $25,000
◯ $25,000 through $49,999
◯ $50,000 through $99,999
◯ $100,000 through $149,999
◯ $150,000 through $199,999
◯ $200,000 and greater
◯ Prefer to not answer

114) What is your race? Check all that apply.

☐ White
☐ Black or African American
☐ American Indian or Alaska Native
☐ Asian
☐ Pacific Islander
☐ Other
☐ Prefer to not answer

115) Are you Hispanic, Latina, or Spanish origin?

◯ Yes
◯ No
◯ Prefer to not answer

You have completed section 7 of 7.

Please double check that you answered all questions before moving to the final page of the survey.
