# Supplementary material for: Association of provider advice and gestational weight gain in twin pregnancies: a cross-sectional electronic survey
Source: BMC Pregnancy Childbirth. 2020 Jul 23;20:417. doi: 10.1186/s12884-020-03107-3 (PMC7376962; doi:10.1186/s12884-020-03107-3)
Supplement: Supplementary file 2 — Additional file 2: Supplementary File 2. Association of provider advice with women’s compliance to the Institute of Medicine provisional GWG guidelines for twin pregnancies, using a fixed GWG in the first trimester and weekly rate of GWG in the second and third trimesters (N = 276). This table displays results from multinomial regression analyses examining associations of women’s report of provider advice on GWG with women’s compliance to the provisional GWG guidelines for twin pregnancies using a secondary approach to categorize GWG. In these analyses, we used a fixed GWG in the first trimester and weekly rate of GWG in the second and third trimesters to categorize GWG as below, within, or above guidelines. [file 12884_2020_3107_MOESM2_ESM.docx]

**Supplemental File 2:** Association of provider advice with women’s compliance to the Institute of Medicine provisional GWG guidelines for twin pregnancies, using a fixed GWG in the first trimester and weekly rate of GWG in the second and third trimesters (N=276)

|  | **GWG Below IOM Guidelines** | | **GWG Above IOM Guidelines** | |
| --- | --- | --- | --- | --- |
| **Provider Advised GWG** | **Adjusted OR**^a^ | **95% CI** | **Adjusted OR**^a^ | **95% CI** |
| Below IOM guidelines | **6.82** | **3.02, 15.44** | 1.36 | 0.55, 3.39 |
| Above IOM guidelines | 0.69 | 0.08, 6.24 | **4.31** | **1.39, 13.34** |
| Within IOM guidelines | Reference | Reference | Reference | Reference |
| Did not discuss | **2.73** | **1.29, 5.80** | 1.83 | 0.92, 3.66 |

Abbreviations: GWG = gestational weight gain, IOM = Institute of Medicine

^a^Model adjusted for maternal age at delivery, education, parity, twin type (dichorionic/diamniotic vs. dichorionic/monoamniotic or monochorionic/monoamniotic), assisted reproductive technologies (yes/no), and pre-pregnancy BMI category. Bolded values are statistically significant (p<0.05).
